# The Hierarchical Process of Differentiation of Long-Lived Antibody-Secreting Cells Is Dependent on Integrated Signals Derived from Antigen and IL-17A

**DOI:** 10.1371/journal.pone.0074566

**Published:** 2013-09-18

**Authors:** Lidiane Zito Grund, Monica Lopes-Ferreira, Carla Lima

**Affiliations:** Immunoregulation Unit, Special Laboratory of Applied Toxinology (CEPID/FAPESP), Butantan Institute and Department of Immunology, University of São Paulo, São Paulo, Brazil; Tulane University, United States of America

## Abstract

Switched CD19-positive memory B cells purified from mice with chronic immune response against *Thalassophryne* *nattereri* venom proteins were cultured with venom or cytokines. Our results confirm the existence of a hierarchic process of differentiation: activated memory B cells progressively acquire increasing levels of CD138 and decreasing levels of CD45R/B220 to finally arrive at ASC with B220^neg^ phenotype, which are IgG1-secreting cells. Only Bmem from peritoneal cavity or bone marrow of V*Tn* immunized mice presented the capacity to generate ASC functionally active. IL-17A or IL-21/IL-23/IL-33 improves the ability of venom to induce intracellular IgG of peritoneal derived-ASC. Cognate stimulation with venom and IL-17A is sufficient to down-regulate the expression of CD45R/B220. BAFF-R is up-regulated in splenic or medullar derived-ASC stimulated by venom, CpG or cytokines. Only splenic derived-ASC up-regulate Bcl-2 expression after CpG or the combination of IL-21/IL-23/IL-33 stimulation. Finally, the activation of ASC for IgG1 secretion is triggered by venom proteins in peritoneal cavity and by IL-17A in medullar niche. These results show the importance of the integration of signals downstream of BCR and IL17-A receptors in modulating ASC differentiation, focusing in the microenvironment niche of their generation.

## Introduction

Immunological memory is typically established following immunization or infections, and is central to the survival of the host. This immunity is engendered by cellular (CD4 and CD8 T cells) and humoral (B cells) immune compartments. Two B cell populations are responsible for sustaining the humoral immune memory: memory B cells (Bmem) and the long-lived antibody-secreting cells (ASC) [1,2,3].

The non-proliferating ASC secrete high affinity antigen-specific antibodies (Abs) for protracted periods of time [1,4], are capable of homing to bone marrow (BM) via CXCR4/CXCL12-mediated chemokine signaling or inflamed tissue and differ from Bmem in many respects. ASC up-regulate Blimp-1, XBP-1, IRF4 that cause i) cessation of cell cycle; ii) decrease signaling from the B cell-receptor (BCR) and communication with T cells; iii) inhibition of isotype switching and somatic hypermutation; iv) down-regulation of CXCR5; v) induction of copious immunoglobulin (Ig) synthesis and secretion; vi) down-regulation of typical B cell markers, including major histocompatibility (MHC) class II, B220/CD45, CD19, CD21, CD22, and surface Ig; vii) and increase of syndecan-1 (CD138) [5,6].

Conventional models suggest that long-term Ab responses are maintained by the continuous proliferation and differentiation of Bmem into ASC. Despite some studies carefully mapping out the mechanisms mediating the survival of Bmem, Hikida et al. [7] report that phospholipase C (PLC)-γ2 is required for efficient formation of germinal center (GC) and Bmem. However, it was described that BAFF and APRIL are not required for the survival [8]. Also it is not clear whether antigen reencounter results in the activation of antigen-responding Bmem or if intrinsic changes modulate their differentiation into ASC following appropriate stimulation [9]. It has been proposed that long-lasting B cell–mediated immunity is sustained by recurrent antigen exposure and in the absence of cognate antigen, inflammatory stimuli associated with adaptive immune responses like cytokines, Toll-like receptor (TLR) agonists or T cell help drive the activation of Bmem in an non-specific manner *in vivo* [10,11]. Signals influencing the decision between memory maintenance and plasmacytic differentiation are not fully understood at present.

Recently, using venom proteins of *Thalassophryne* *nattereri* (V*Tn*) Brazilian fish we establish a model in which GC derived-B cells and high-affinity specific Abs were permanently generated [12]. Therefore, this model provides an interesting scenario for studying the signals allowing survival and differentiation of the memory B cell compartment. In particular, humoral memory response to venom was characterized by a predominant production of IgG2a Abs that decline after 74 d privileging the production of IgE Abs later (120 d). A chronic expansion of B1a cells in BM induced by the venom was also observed, splenic cells retained venom proteins and in the peritoneal cavity a Th2-mediated inflammation with infiltration of eosinophils, mast cells, neutrophils and IL-17A-producing CD4+ CD44+ CD40L+ Ly6C+ effector memory T cells (TeM) were maintained. The venom promoted the differentiation of Bmem and subtypes of ASC that were characterized by the expression of B220 and CD43 molecules (B220 ^high^CD43^high^, B220 ^high^CD43^low^, B220 ^low^CD43^high^ or B220 ^neg^CD43^high^), indicating a hierarchical process of differentiation [13].

Furthermore, we have provided *in vivo* evidence that IL-17A as well as IL-5 produced in a context of chronic inflammatory response against venom proteins directly influence the production of specific IgE Abs and the maintenance of B1a cells in the BM from the spleen. Both cytokines negatively regulate the maintenance of ASC B220^pos^ in different sites of response. A striking finding in this study was that IL-5 and IL-17A are critical for the differentiation and maintenance of ASC B220^neg^ phenotype in inflamed peritoneal cavity [13].

Here in this study, we proposed to confirm the capacity of memory B cells generated by venom proteins to undergo terminal differentiation in response to different immunological signals as re-exposition of antigen or non-specific and bystander mediators as cytokines.

## Material and Methods

### Venom

*Thalassophryne* *nattereri* fish venom was obtained from fresh captured specimens in different months of the year according to Lopes-Ferreira et al. [14] at the Mundau Lake in Alagoas, state of Brazil with a trawl net from the muddy bottom of lake. No protected specimens were captured and fish were transported to Immunoregulation Unit of Butantan Institute. All necessary permits (capture, conservation and venom c) were obtained for the described field Studies (Instituto Brasileiro do Meio Ambiente e dos Recursos Naturais Renovaveis - IBAMA Permit Number: 16221-1). Venom was immediately extracted from the openings at the tip of the spines by applying pressure at their bases. After that fish were anesthetized with 2-phenoxyethanol prior to sacrifice by decapitation.

After centrifugation, venom was pooled and stored at -80 °C before use. The venom protein concentration was determined by the Bradford [15] colorimetric method using bovine serum albumin as the standard (Sigma Chemical Company; ST. Louis, MO, USA). Endotoxin content was evaluated (resulting in a total dose < 0.8 pg/mL LPS) with QCL-1000 chromogenic *Limulus* *amoebocyte* lysate assay (Bio-Whittaker) according to the manufacturer’s instructions.

### Mice

Male BALB/c mice (5–6 weeks old) were obtained from a colony at the Butantan Institute, São Paulo, Brazil. Mice were housed in a laminar flow holding unit (Gelman Sciences, Sydney, Australia) in autoclaved cages on autoclaved bedding, in an air-conditioned room in a 12 h light/dark cycle. Irradiated food and acidified water were provided *ad libitum*. This study was carried out in strict accordance with the recommendations in the Guide for the Care and Use of Laboratory Animals of the Brazilian College of Animal Experimentation. The protocol was approved by the Committee on the Ethics of Animal Experiments of the Butantan Institute (Permit Number: 666/09) and of University of São Paulo (Permit Number: 25/84/02). All surgery was performed under sodium pentobarbital anesthesia, and all efforts were made to minimize suffering.

### Induction of memory immune response by venom

Groups of 5 mice were immunized with intraperitoneal (i.p.) injections of 10 µg of *Thalassophryne* *nattereri* fish venom on days 0 and 14. The first immunization was give in 1.6 mg of aluminium hydroxide (Al(OH)_3_) as adjuvant and the booster in the absence of adjuvant. Mice injected only with Al(OH)_3_ were considered as control-group. After 48 d, mice were killed by injection of lethal dose of sodium pentobarbital anesthesia for obtaining peritoneal, spleen and BM cell suspensions.

### Peritoneum, splenic and bone marrow cell isolation

Cell suspensions from control or immunized mice were obtained at 48 d after the first immunization. Peritoneal cells were recovered by peritoneal lavage using 5 mL of ice-cold sterile phosphate-buffered saline (PBS) plus 0.1% EDTA (ethylenediaminetetraacetic acid). Spleens were dissociated into single cell suspensions by mechanical disruption in Cell Strainer (BD Falcon). Bone marrow cells were obtained by flushing femurs of mice. Erythrocytes in spleens and BM were lysed with 0.14 M NH_4_Cl and 17 mM Tris-HCl (pH 7.4). After lyses, cell concentration was adjusted to 10 x 10^6^ cell/mL in RPMI containing 10% heat-inactivated FCS.

### CD19-positive memory B cell purification

B cells were purified from either control- or V*Tn*-immunized BALB/c (48 d) mice using Magnetic Activated Cell Sorting (MACS, Miltenyi Biotec, Bergisch Gladbach, Germany). A single-cell leukocyte suspensions from freshly isolated spleen, bone marrow, and the peritoneal cavity were prepared using RPMI containing 10% heat-inactivated FCS. Erythrocytes were removed from the single cell suspensions by lysis. Briefly, total cells (1 × 10^7^) were incubated with 10 µL of anti-CD19 (Ly-1) MicroBeads (Miltenyi Biotec) according to the manufacturer’s instructions for positive selection. After immobilization of all these cells with a magnet, untouched cells were discharged and CD19-positive B cells were collected and identified. Purity of Bmem cells identified as CD19^+^ was 95% and confirmed by flow cytometry.

### CD19-positive memory B cell culture

All cultures were performed in Iscove modified Dulbecco medium (Invitrogen) and 10% fetal calf serum. Purified CD19-positive B cells from peritoneum, spleen and BM were plated at 1.5 x 10^5^/mL and cultured in basic conditions that favors B differentiation according to Jourdan et al. [16]. In the first step of activation (0-4 d) B cells were cultured in the presence of soluble anti-CD40 mAB (50 ng/mL) and recombinant cytokines as IL-2, IL-4 and IL-10 (all at 50 ng/mL). In respective cultures group, 2.5 µg/mL of CpG-ODN (*oligodeoxynucleotide* 24, Sigma-Aldrich) or *T* *. nattereri* venom (20 µg/mL) were added. After 4 d of culture, plasmablast were harvested, washed, and cultured with IL-2, IL-10 and IL-6 (all at 50 ng/mL) or with various combinations of recombinant cytokines as IL-17A, IL-21, IL-23 and IL-33 (all at 1 ng/mL). At 7 d of culture, cells were washed and cultured with recombinant IL-6 (50 ng/mL) for 2 d for plasma cell generation.

### Detection of apoptosis or necrosis

Apoptotic and necrotic cells were analyzed with an FITC-Annexin V (*fluorescein isothiocyanate* FITC)-conjugated or PI using flow cytometry. Cells were harvested and resuspended in 100 µL binding buffer. Subsequently, cells were incubated with 5 µL of FITC-Annexin V and 10 µL of PI for 15 min in the dark. The intensity of fluorescence of stained cells was acquired using a BD FACSCalibur flow cytometer and data were analyzed with CellQuest software (BD Biosciences, Mississauga, ON, Canada).

### Labeling with CFSE

For monitoring cell division, B cells in the first day and in the last day of culture (1 x 10^6^ cell/mL) were incubated for 10 min at 37 °C with 5 mM CFSE (*5- and 6-carboxyfluorescein diacetate succinimidyl ester*; Molecular Probes). After being washed extensively, cells were resuspended in culture medium and cell proliferation was measured on day 4 by flow cytometry on a FACSCalibur and data were analyzed with CellQuest software (BD Biosciences). A combination of CFSE and PerCP-Cy5-anti-mouse CD45R/B220 or PE-anti-mouse CD138 was used to determine B cell differentiation status before and after culture.

### Hematoxilin/eosin staining

The CD19-positive B cell pellets before and after culture were resuspended in PBS containing 0.1% newborn calf serum (Sigma) and slides were performed using a hemocytometer and cytocentrifuge. Slides were air dried, fixed in methanol, and stained (Wright-Giemsa, Scientific Products, Chicago, IL). After wash in H_2_O they were mounted for observation with light microscopy at a magnification of ×40 (Axio Imager A1; Carl Zeiss).

### Flow Cytometry Analysis

For surface staining single-cell suspensions (1 x 10^6^) were treated with 3% mouse serum of naive mice to saturate Fc receptors followed by the staining by fluorescence conjugated Abs: Rat IgG2ak PE-anti-mouse CD138, Rat IgG2ak PerCP-Cy5-anti-mouse CD45R/B220, Rat IgG2ak FITC-anti-mouse CD19 and Rat IgG2bk FITC-anti-mouse BAFF-R for 30 min in ice. Cells were washed three times in PBS 1% BSA. For intracellular staining, cells were washed, fixed and permeabilized with Cytofix/Cytoperm solution (BD Biosciences) and stained with Rat IgG2ak PerCP-Cy5-anti-mouse Bcl-2 and Rat IgG2bk FITC-anti-mouse IgG. Cells were washed three times in PBS 1% BSA. Negative-controls were used to set the flow cytometer photomultiplier tube voltages, and single-color positive controls were used to adjust instrument compensation settings. Cells were examined for viability by flow cytometry using side/forward scatter characteristics or 7-AAD exclusion. Data from stained samples were acquired using a four-color FACSCalibur flow cytometer equipped with CellQuest software (BD Biosciences) and were analyzed using CellQuest Software (Becton-Dickinson, San Jose, CA). Data were recorded as geometric mean fluorescence intensity (MFI) and percent of fluorescent positive cells.

### Determination of IgG production

The concentration of venom-specific IgG in cell culture supernatants was measured on day 9 with quantitative ELISA. Supernatants were tested for IgG1 or IgG2a Abs using venom-coated 96-well plates (venom at 3 µg/mL) and biotinylated goat anti-mouse IgG1 or IgG2a antiserum. The reactions were developed with streptavidin-horseradish peroxidase complex (Sigma), OPD (O-phenylenediamine) and H_2_O_2_ and plates were read at 490 nm on an automated ELISA reader (Spectramax, Molecular Devices). Results were expressed as the mean ± SEM absorbance. Antibody concentrations were calculated from the IgG standard curves and represented as µg/mL.

### Statistical analysis

All values were expressed as mean ± SEM. Parametric data were evaluated using an analysis of variance, followed by the Bonferroni test. Non-parametric data were assessed using the Mann–Whitney test. Differences were considered statistically significant at *p* < 0.05. The SPSS statistical package (Release 13.0, Evaluation version, 2004) was employed.

## Results

### Memory response induced by *T* *. nattereri* venom is characterized by high frequency of CD19-positive Bmem

In our previously study [13] we identified that proteins of V*Tn* induce in BALB/c mice a chronic humoral response characterized by the presence of Bmem and ASC in peritoneum, spleen and BM at several time-points after immunization. Also we demonstrated that 48 d post-immunization was a time for high frequency of switched Bmem (CD45R/B220 ^pos^IgG ^pos^CD19^pos^) and low frequency of ASC (CD45R/B220 ^neg^CD138^pos^) in all three compartments: 2.9% control vs 87.5% V*Tn* in peritoneal cavity, 10% control vs 71% V*Tn* in spleen, and 10% control x 79% V*Tn* in bone marrow (Figure S1), thus becoming an ideal period for purifying cells committed with terminal B cell differentiation. Second, B cell-restricted cell surface protein CD19 has been used as a good murine marker of naive, activated and memory B cell that appears at the earliest stages of development [17] but is down-regulated during plasma cell differentiation [18].

**Figure 1:**
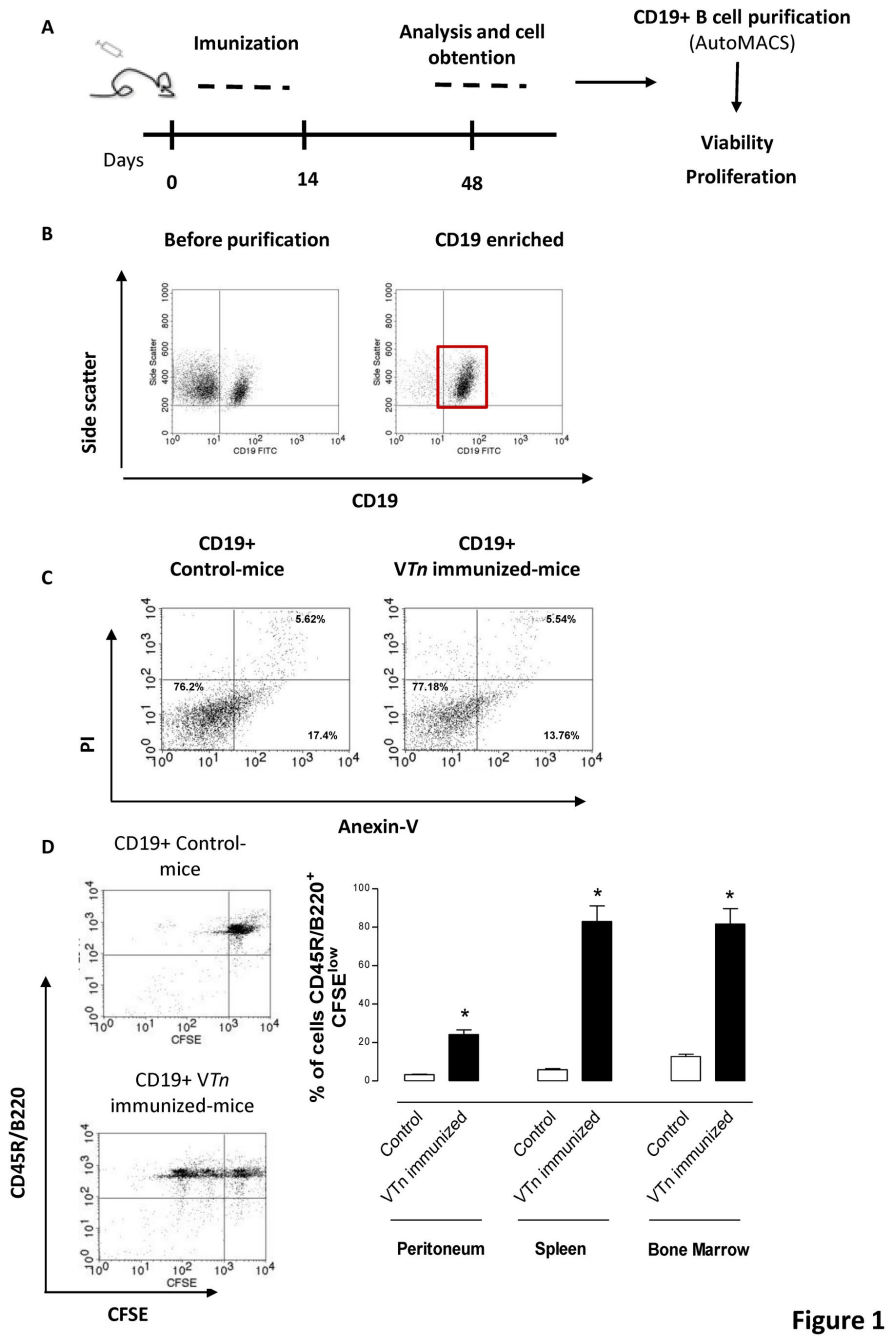
Memory response induced by *T* *. nattereri* venom is characterized by high frequency of CD19-positive B cells. Cartoon show the course of the experimental protocol in BALB/c mice immunized i.p. with 10 µg of *T* *. nattereri* venom (V*Tn*) adsorbed in Al(OH)_3_ on days 0 and 14. Mice injected only with Al(OH)_3_ were considered as control group. After 48 d, mice were killed for peritoneal, spleen and BM cell suspensions collection. CD19-positive cells were enriched using magnetic anti-CD19 microbeads and positive selection (*A*). Purity (*B*) and viability (*C*) were assessed by flow cytometry using CD19 staining and FITC-annexin V co-staining with propidium iodide (PI) respectively. The percentage of cells in proliferation was determined through CFSE incorporation. The percentage of CD45R/B220^pos^ CFSE^low-^labeled CD19-positive B cells from control- or V*Tn*-immunized mice was assessed by flow cytometry after 4 d of culture (*D*). Data are mean ± SEM values from three independent experiments. *p < 0.05 compared to CD19-positive B cells from control. Dot plots are representative of 3 experiments.

Then we choose this period of time (48 d) to purified CD19-positive B cells using magnetic microbeads (Figure **1A**). Several protocols sorting human memory B cells that are committed to plasmacytic differentiation use CD27 as well as CD19 molecule. Here we purified CD19-positive switched memory B lymphocytes from V*Tn*-immunized mice and CD19-positive naive B cells from control-mice.

We confirmed the enrichment process of CD19-positive B cell by positive selection (Figure **1B**) in association with a high percentage of viable cells (control- 76.98% vs V*Tn*-immunized mice 80.70%) (Figure ***1C***). We also showed that only CD19-positive B cells derived from V*Tn*-immunized mice proliferate *in vitro*, compared with the low capacity of proliferation of CD19-positive B cells from control mice, indicative of the existence of naive B cells in control-mice and effector/memory B cells in venom-mice. The high proliferative response (16-fold) was achieved using splenic CD19-positive B cells from V*Tn*-immunized mice, followed by high frequency of BM and peritoneal cells (Figure **1D**).

Together, these results show that 48 d after *in vivo* V*Tn*-immunization, the venom proteins are able to induce viable effector/memory CD19-positive B cells, particularly in spleen, with a proliferative capacity in medium without any specific stimulation. In humans, approximately one third of the CD19-positive B cells is Bmem on a average basis [19]. Traditionally, the induction of Bmem is considered as a crucial factor for long-term vaccine-induced protection [10,11]. The high frequency achieved in our model upon venom immunization is similar with frequencies observed in humans by components of bacterial vaccines (*Bordetella pertussis* and tetanus) or viral vaccines (measles and influenza) [20].

### CD19-positive Bmem generated by VTn differentiate in vitro into non-proliferating CD138-positive ASC

**Figure 2:**
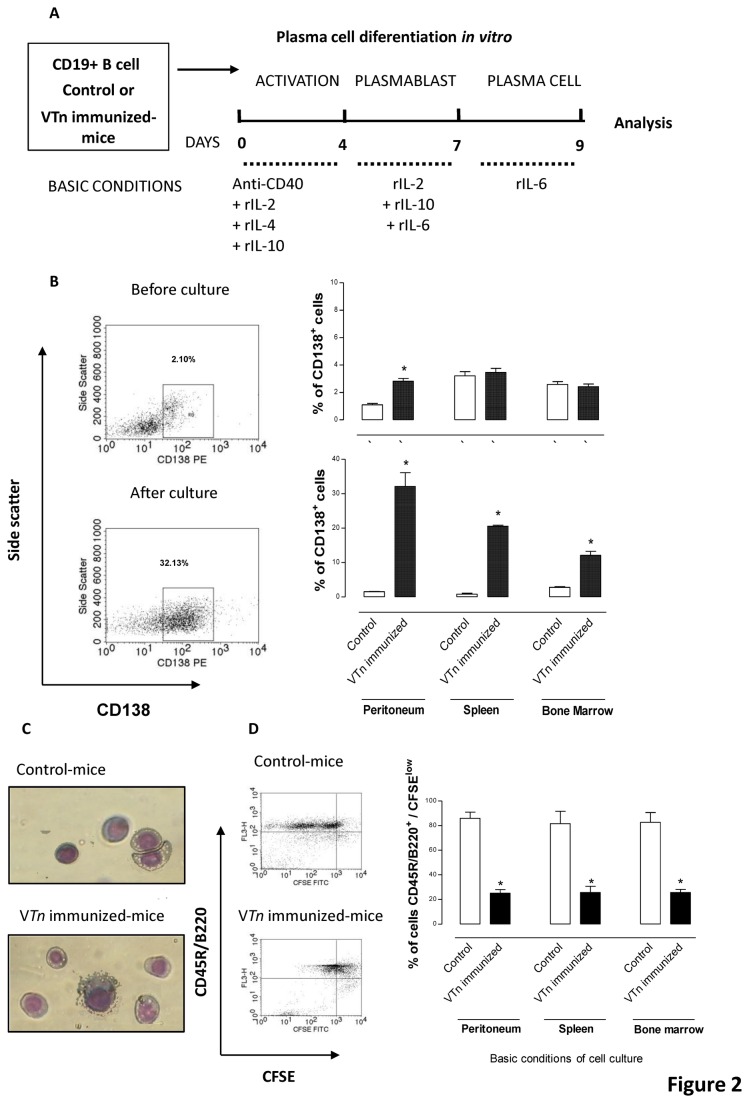
Venom is able to induce the *in vitro* differentiation of CD19-positive Bmem into non-proliferating CD138-positive ASC. Representation of the B cell differentiation in an *in* *vitro* model (*A*). Purified CD19-positive Bmem (1.5 x 10^5^ cell/mL) obtained 48 d after venom immunization were cultured under basic conditions to plasma cell generation for 9 d. ASC differentiation was phenotypically monitored by flow cytometry based on CD138 membrane expression (*B*) and morphologically by Hematoxilin/eosin staining (*C*). The percentage of proliferating-cells was determined through CFSE incorporation. CD138^pos^ CFSE^low-^labeled CD19-positive B cells from control- or V*Tn*-immunized mice were assessed by flow cytometry after 4 d of culture (*D*). Data are mean ± SEM values from three independent experiments. *p < 0.05 compared to CD19-positive B cells from control. Dot plots are representative of 3 experiments.

Next we investigated the commitment of Bmem to plasmacytic differentiation (ASC) and if there is a linear process using an *in vitro* system. For that, purified CD19-positive B cells (1.5 x 10^5^ cell/mL) from control- immunized mice (naive B cells) or V*Tn*-immunized mice (memory B cells) were cultured in a three-step in *vitro* model with medium under basic conditions to B cell maintenance and differentiation for 9 days according to procedure schematized in detailed on Figure **2A**. ASC differentiation was confirmed by the CD138 membrane expression acquisition and loss of their proliferative capacity. CD138 was reported to be expressed in ASC in BM and peripheral blood, but not on pre-germinal centre B cells [21]. CD138 is a heparan sulphate proteoglycan, which mediates cellular adhesion to collagen type I [22] and might play a role in adhesion to BM stromal cells [23].

In Figure **2B** we see that before culture (upper) only CD19-positive B cells purified from peritoneum of V*Tn*-immunized mice express high levels of CD138 compared with CD19-positive B cells from control group. After culture (bottom) in medium under basic conditions, cells obtained from all compartments mainly peritoneal and splenic CD19-positive B cells from V*Tn*-immunized mice up-regulated the expression of CD138 after differentiation.

Next we confirm the status of terminal differentiated CD138-positive ASC from V*Tn*-immunized mice. In Figure **2C** we show that ASC from V*Tn*-immunized mice (lower picture) presented an activated lymphocyte-like morphology reminiscent of plasma cell with a small, dense, ovoid nucleus and a voluminous cytoplasm containing prominent amounts of rough endoplasmic reticulum (RER) and enlarged Golgi compared with naive B cells from control mice that exhibit a high nucleus to cytoplasm ratio, little RER, and an uncondensed nucleus (upper picture). According to CFSE staining (Figure **2D**), after culture in basic conditions, only few cells of V*Tn*-immunized mice are dividing, confirming the loss of the capacity of proliferation after stimulation (black bars - Figure **2D**). On the other hand, CD19-positive B cells purified of all cell suspensions obtained from control mice show a great proliferative capacity under basic condition of culture (white bars - Figure **2D**).

Here, these findings confirm the existence of a hierarchic process of differentiation in which CD19-positive Bmem from mice with chronic response to the venom differentiate *in vitro* into CD138-positive ASC. Terminal differentiated ASC express high levels of CD138 and posses low proliferative capacity.

### IL-17A and a combination of IL-21/IL-23/IL-33 potentiate the effect of IgG production induced by venom

Early studies demonstrated that IL-17A participates on antigen-specific Ig production since the efficient levels of Ig were reduced in mice deficient in IL-17 [24]. Some mediators as IL-21 cytokine not only trigger B-cell proliferation [25], isotype switching and somatic hypermutation [26], but also induce ASC differentiation, exceeding 5 to 20 times the capacity of IL-4, IL-2 or IL-10 in this function [27]. IL-33 has been described by increase IgG1 and IgG2a production in inflammatory diseases such as collagen-induced arthritis [28] and recently, IL-23R was detected in plasmacytes and plasmablasts and the signals derived modulate IgM and IgG secretion [29].

**Figure 3:**
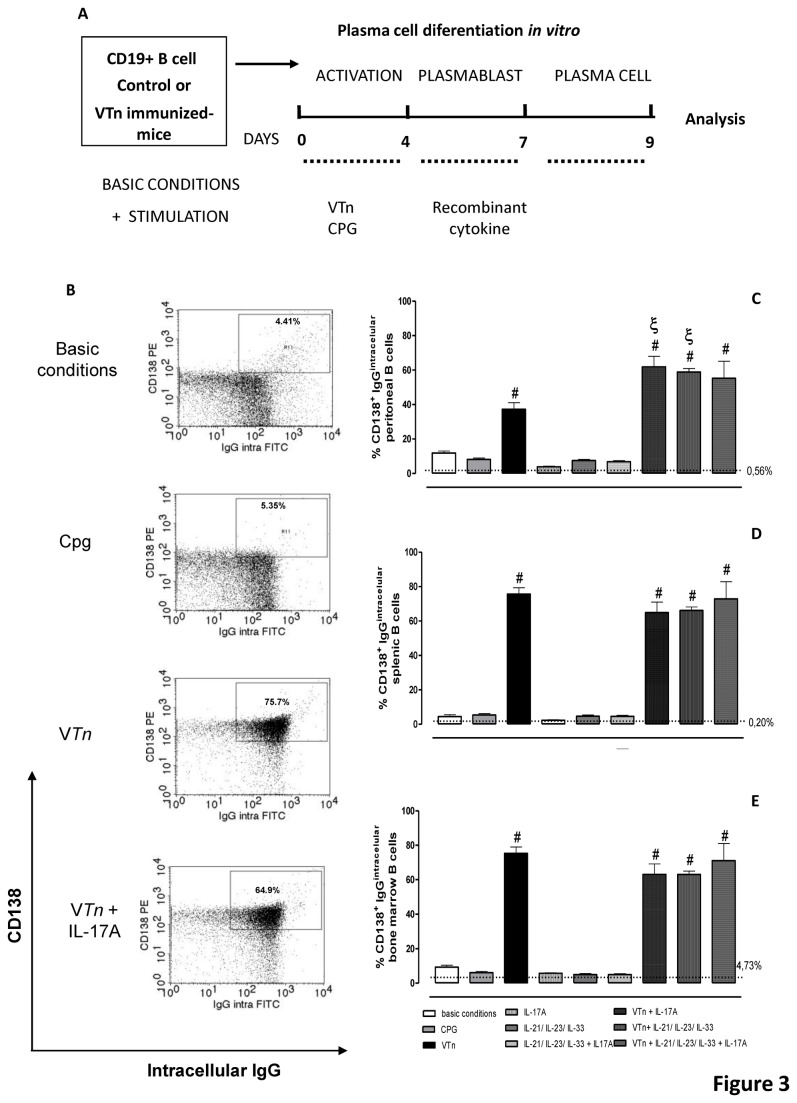
IL-17A and a combination of IL-21/IL-23/IL-33 potentiate the ability of venom to induce the differentiation of IgG producing-ASC. Representation of the B cell differentiation in an *in* *vitro* model. Purified CD19-positive Bmem (1.5 x 10^5^ cell/mL) obtained 48 d after venom immunization were cultured in a three-step in *vitro* model under basic conditions or in medium supplemented with V*Tn*, CpG or cytokines alone or in combination with venom for 9 d (*A*). Evaluation of intracellular content of IgG in CD138-positive ASC was determined by flow cytometry (*B*). The percentage of double-positive cells was analyzed in peritoneal (*C*), splenic (*D*) or medullar cells (*E*). The dashed line represents the percentage of IgG^pos^ CD138^pos^ ASC differentiated from CD19-positive B cells from control group of mice cultured in medium under basic conditions. #*p* < 0.05 compared to CD19-positive B cells from V*Tn*-immunized mice in medium under basic conditions; and ξ*p* < 0.05 compared to CD19-positive B cells from V*Tn*-immunized mice in medium supplemented with V*Tn*. Data are mean ± SEM values from three independent experiments. Dot plots are representative of 3 experiments.

To gain insight into extrinsic cues required for ASC differentiation and reinforce the hierarchical process of differentiation of Bmem into ASC, we evaluated the role of the venom antigens and the co-participation of recombinant cytokines or CpG in this culture system (Figure **3A**). Because ASC lose their ability to cell division, reduce the expression of genes involved in BCR signaling and over-express genes involved with Ig production, we analyze after 9 d of culture the percentage of double positive cells: CD138-positive IgG producing-ASC (Figure **3B**). These results show that V*Tn* re-stimulation *in vitro* enhances the percentage of CD138-positive IgG producing-ASC from cells of the all compartments of immunized mice; in contrast with the incapacity of unrelated antigen as CPG (Figure 3C-3E).

These findings suggest an antigen-specific process and corroborate the idea that the differentiation of Bmem into ASC during T-dependent responses is at least in some cases strictly dependent on their expression of MHC-II [30].

The recombinant cytokine IL-17A as well as the combination of IL-21/IL-23/IL-33 cytokines have additive effect on peritoneal ASC differentiation induced by V*Tn*. However, the addition of IL-17A or the combination of cytokines IL-21/IL-23/IL-33 did not play a synergic effect on splenic or BM ASC differentiation induced by V*Tn*. Such event may be explained by the *in vivo* microenvironment in which splenic and BM cells developed. After 48 d of immunization with V*Tn* we detect the production of large amounts of IL-17A in all compartments including peritoneal cavity, but IL-10 was produced only by splenic and BM cells [13]. The presence of IL-17A could up-regulate the expression of IL-17R in the CD19-positive Bmem while IL-10 could counter-regulate this expression. So, we can speculate that peritoneal Bmem expressing high levels of IL-17R could be more susceptible to *in vitro* action of IL-17A, in contrast to BM and splenic cells that are more refractory to this signal.

Also, TLR9 agonist, the mixture of IL-21/IL-23/IL-33 alone, IL-17A alone or added to IL-21/IL-23/IL-33 mixture did not directly induce ASC differentiation from cells of any compartment (Figure **3C-3E**).

Our results together confirm the existence of a hierarchical process in which CD19-positive Bmem become CD138-positive IgG producing-ASC by a mechanism directly dependent on BCR stimulation by venom, that could be potentiated by IL-17A and IL-21/IL-23/IL-33 if the cells are from peritoneal cavity.

### Loss of CD45R/B220 surface expression in ASC is controlled by cognate antigen

**Figure 4:**
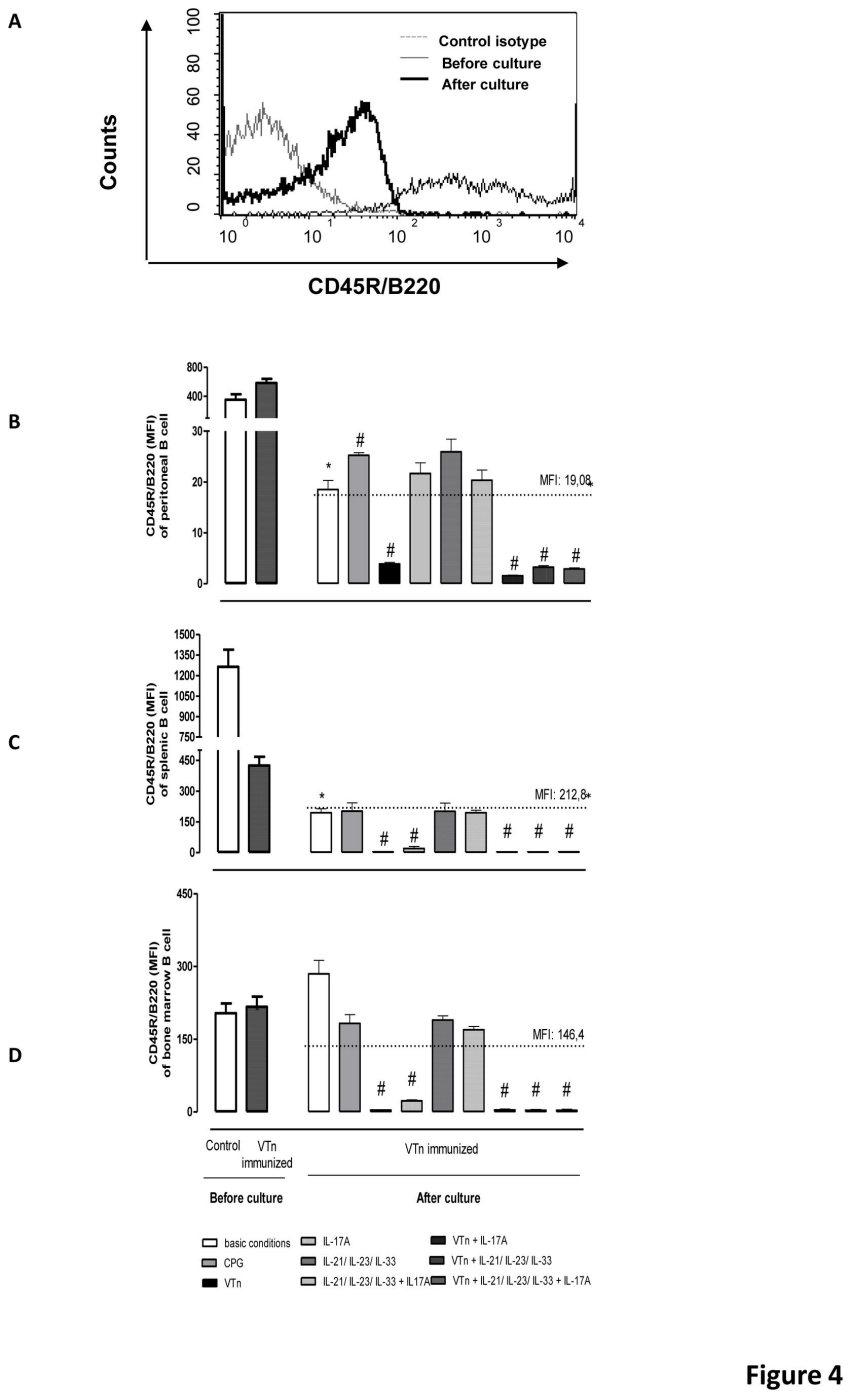
Loss of CD45R/B220 surface expression in ASC is controlled by cognate antigen. The surface expression of CD45R/B220 was analyzed in terms of mean fluorescence intensity (MFI) ± SD by flow cytometry in CD138-positive ASC derived from CD19-positive B cells of control- or V*Tn*-immunized mice. Histogram is representative of 3 experiments (*A*). The dashed line represents the MFI of CD45R/B220 in purified CD19-positive B cells from control mice cultured in medium under basic conditions. The percentage of positive cells was analyzed in peritoneal (*B*), splenic (*C*) or medullar cells (*D*). *p < 0.05 compared to CD19-positive B cells from control, and #*p* < 0.05 compared to CD19-positive B cells from V*Tn*-immunized mice in medium under basic conditions.

CD45R/B220 glycoprotein is a member of the family of protein tyrosine phosphatases expressed in B lymphocytes throughout their development from early pro-B stages and is down-regulated upon terminal differentiation into ASC [31]. Decreased expression of CD45R/B220 is particularly significant for ASC longevity since its lack increases cell survival [32]. Our next step was the evaluation of the expression of the CD45R/B220 in ASC differentiated from Bmem collected of V*Tn*-immunized mice (Figure **4**).

First, we noted that before culture, the peritoneal (Figure **4B**) and BM (Figure **4D**) CD19-positive B cells from V*Tn* (gray) or control mice (white) express similar levels of CD45R/B220, in contrast to splenic (Figure **4C**) CD19-positive B cells from V*Tn*-immunized mice that presented lower expression compared with cells from control mice. This result suggests that the *in vivo* splenic microenvironment selectively controls the low levels of CD45R/B220 expression.

Second, after 9 d of basic conditions, peritoneal (Figure **4B**) and splenic (Figure **4C**) differentiated ASC from Bmem of V*Tn*-immunized mice showed decreased CD45R/B220 levels, while BM cells (Figure **4D**) maintain similar levels compared with before culture of this molecule. When Bmem of V*Tn*-immunized mice were re-stimulated *in vitro* with GpG we observed that this TLR9 agonist up-regulated the expression of CD45R/B220 only in peritoneal ASC, but did not change the expression in splenic or medullar ASC.

The re-stimulation with V*Tn* dramatically decreased the CD45R/B220 expression in ASC from Bmem of all compartments, whereas IL-17A alone only induced decrease in CD45R/B220 levels in ASC from splenic and medullar niche. The addition of the mixture of 3 or 4 cytokines to peritoneal, splenic or medullar Bmem was not able to induce decrease in the CD45R/B220 expression levels in differentiated ASC. Also, the addition of cytokines (mixed of 3 or 4 cytokines) to culture re-stimulated with V*Tn* did not enhance the venom capability of decrease the CD45R/B220 expression in ASC. These results show that although IL-17A plays co-participating with V*Tn* in the differentiation of peritoneal Bmem into IgG producing CD138-positive ASC, probably due to its ability to induce increased expression of IL-17R, this cytokine alone is not sufficient to decrease CD45R/B220 expression in peritoneal cells, suggesting a direct requirement of V*Tn* and others signaling pathways on peritoneal Bmem for down-regulation of CD45R/B220. For example, the classical XBP-1/Blimp-1 dependent pathway [6]. IRF-4, Blimp-1 and XBP-1/UPR transcriptional regulators are important in the control of the terminal differentiation of memory B lymphocytes into ASC [33].

### ASC from splenic and medullar CD19-positive B cell express high levels of BAFF-R

**Figure 5:**
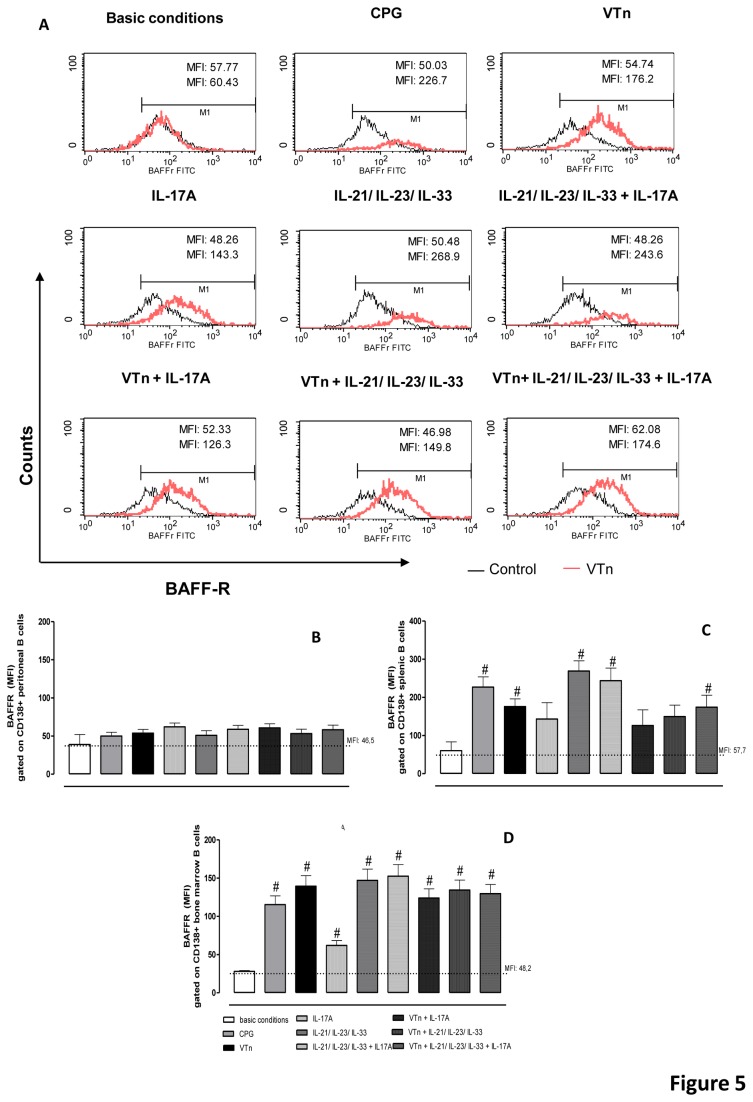
ASC from splenic and bone marrow CD19-positive B cells express high levels of BAFF-R. The surface expression of BAFF-R was analyzed in terms of mean fluorescence intensity (MFI) ± SD by flow cytometry in CD138-positive ASC derived from CD19-positive B cells of control- or V*Tn*-immunized mice. Histogram is representative of 3 experiments (*A*). The dashed line represents the MFI of BAFF-R in purified CD19-positive B cells from control mice cultured in medium under basic conditions. The percentage of positive cells was analyzed in peritoneal (*B*), splenic (*C*) or medullar cells (*D*). #*p* < 0.05 compared to CD19-positive B cells from V*Tn*-immunized mice in medium under basic conditions.

BAFF (*B cell activating factor*), a member of the TNF family (also named TALL-1, THANK, BlyS or zTNF4) plays a fundamental role in the long-term survival and homeostasis of mature B2 and marginal zone B cells [34]. The binding of BAFF to their receptors (BAFF-R/BR3, TACI, BCMA) leads to the activation of the NF-κB pathway and ultimately to the transcription of the anti-apoptotic factor Bcl-xL and Bcl-2 [35]. We reported in Figure 5A and 5B that CD138-positive ASC differentiated from peritoneal cavity of V*Tn-*immunized mice (white bar) present low levels of BAFF-R similar to the levels of control mice (dashed line). After different types of *in vitro* re-stimulation we observed no changes in the low levels of BAFF-R in ASC, suggesting that another receptors as TACI or BCMA could be required for peritoneal ASC differentiation.

In contrast, our data show that CD138-positive ASC differentiated from spleen of V*Tn*-immunized mice super-express the BAFF-R levels after stimulated with CPG, V*Tn* or the combination of IL-21/IL-23/IL-33 (Figure **5C**). Moreover, added to the capacity of CPG, V*Tn* or the combination of IL-21/IL-23/IL-33 to the up-regulation of the BAFF-R expression, IL-17A is also important for ASC derived from BM cells (Figure **5D**). These findings demonstrated the essential role of BAFF-BAFF-R signaling during ASC differentiation induced by venom in the splenic and BM microenvironment. Other studies clearly demonstrated that splenic follicles are greatly reduced in size, and IgG immune response to T-dependent antigen was impaired as a consequence of anti-BAFF-R blocking Abs [34].

CpG is currently being used as an adjuvant in vaccination protocols [36]. In human B cells, the effects of CpG-ODN-mediated TLR9 activation include cellular proliferation, differentiation into ASC, up-regulation of molecules involved in immune cellular interactions and increase of cytokine secretion. It was recently demonstrated that CpG-ODN induce the expression of TACI and BCMA, but did not up-regulate BAFF-R expression in isolated resting B cells from healthy donors [37]. Here, we demonstrated that TLR9 agonist induced an up-regulation of BAFF-R in ASC from splenic and medullar Bmem of V*Tn*-immunized mice. These results indicate a potential difference in the response of human and murine B cells to CpG-ODN, and show that CpG-ODN synergize with antigen for the induction of increase in BAFF-R expression on murine ASC.

IL-6 and IL-10 [38] are known to be required for proliferation and differentiation of human B cells. Previously we demonstrated that in the memory response induce by V*Tn*, IL-10 was produced only by splenic and BM cells, but not by peritoneal cells [13]. Together we can propose that the up-regulation of BAFF-R in CD138-positive ASC differentiated from spleen and BM of V*Tn-*immunized mice induced by V*Tn*, CPG, or the combination of IL-21/IL-23/IL-33 and IL-17A could require IL-10 co-participation.

### TLR9 agonist and the combination of IL-21/IL-23/IL-33 promote increase in pro-survival Bcl-2 protein in ASC from splenic niche

Terminally differentiated ASC are non-cycling and thus phenotypically different from their predecessors. Expression of Blimp-1 protein results in concomitant repression of the B cell-specific transcription and apoptotic factors as Bcl-6 and Pax5, and up-regulation of pro-survival members of the Bcl-2 family, especially Bcl-2, Bcl-XL and myeloid cell leukaemia 1 (Mcl1) [39]. Over-expression of Bcl-2 also causes a prominent expansion of memory compartment contributing to the maintenance of T and B cell memory [40].

**Figure 6:**
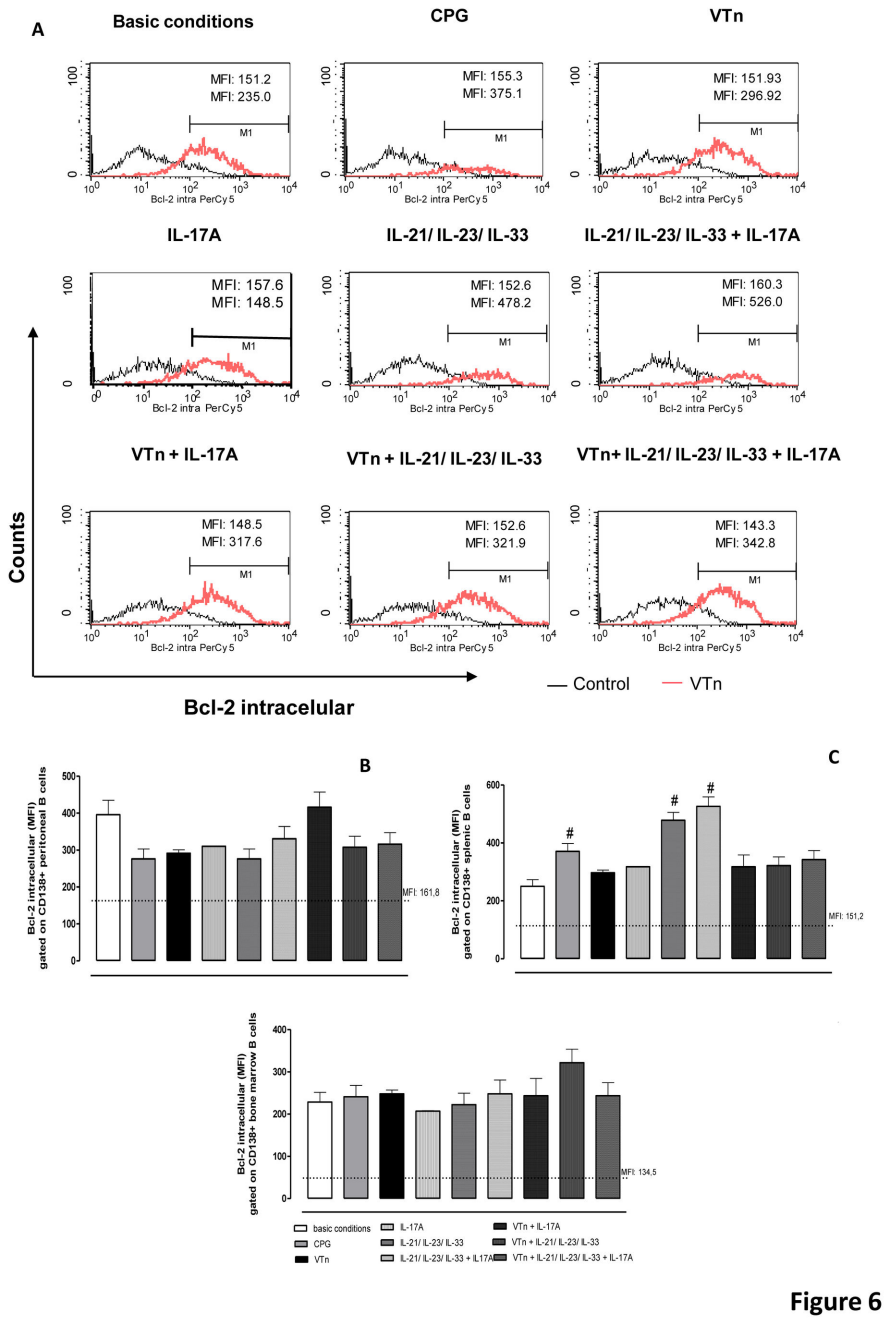
TLR9 agonist and recombinant cytokines promote increase in anti-apoptotic Bcl-2 protein in ASC. The intracellular content of Bcl-2 was analyzed in terms of mean fluorescence intensity (MFI) ± SD by flow cytometry in CD138-positive ASC derived from CD19-positive B cells of control- or V*Tn*-immunized mice. Histogram is representative of 3 experiments (*A*). The dashed line represents the MFI of Bcl-2 in purified CD19-positive B cells from control mice cultured in medium under basic conditions. The percentage of positive cells was analyzed in peritoneal (*B*), splenic (*C*) or medullar cells (*D*). #*p* < 0.05 compared to CD19-positive B cells from V*Tn*-immunized mice in medium under basic conditions.

Our results of intracellular content of Bcl-2 (Figure 6A) show that ASC differentiated from peritoneal (Figure **6B**) or medullar (Figure **6D**) CD19-positive Bmem did not demonstrate up-regulation of Bcl-2 expression after any type of stimulation. But in contrast, only TLR9 agonist (CpG) and the combination of cytokines IL-21/IL-23/IL-33 promote an increase of Bcl-2 expression levels in CD138-positive ASC differentiated from splenic Bmem from V*Tn*-immunized mice (Figure **6C**).

These results corroborate the study of Klein et al. [41] that showed that after leaving the GC, ASC modulate the expression of various genes (267) including Bcl-2 similar to those found in quiescent naive cells. These findings suggest that ASC survival induced by V*Tn* and IL-17A could be mediated by other survival molecules as members of the Rho family GTPases such as Rho, Rac or Cdc42 that regulate the actin cytoskeleton and survival [42].

Moreover our results pointed to an important role for TLR signaling in memory B cell compartment. The key role of TLR receptors in cellular activation and modulation of quality of function of B effector cells was first described by Leadbetter et al. [43]. Our data show that activation of the TLR9 by CpG agonist promotes increased expression of CD45R/B220 in ASC derived from peritoneal B cells (Figure **4B**), of BAFF-R expression in splenic and BM (Figure 5C and 5D) and of Bcl-2 levels by splenic B cells (Figure **6B**). However, the super-regulation of CD5R/B220, BAFF-R and Bcl-2 expression in ASC induced by CpG did not transduce sufficient signals to induce the production or the secretion of specific IgG by ASC. These results suggest that signaling via TLR9 present in endossomal compartments of B cells could be related with ASC survival, but not with Abs production.

### Venom and IL-17A control specific IgG1 secretion by ASC

**Figure 7:**
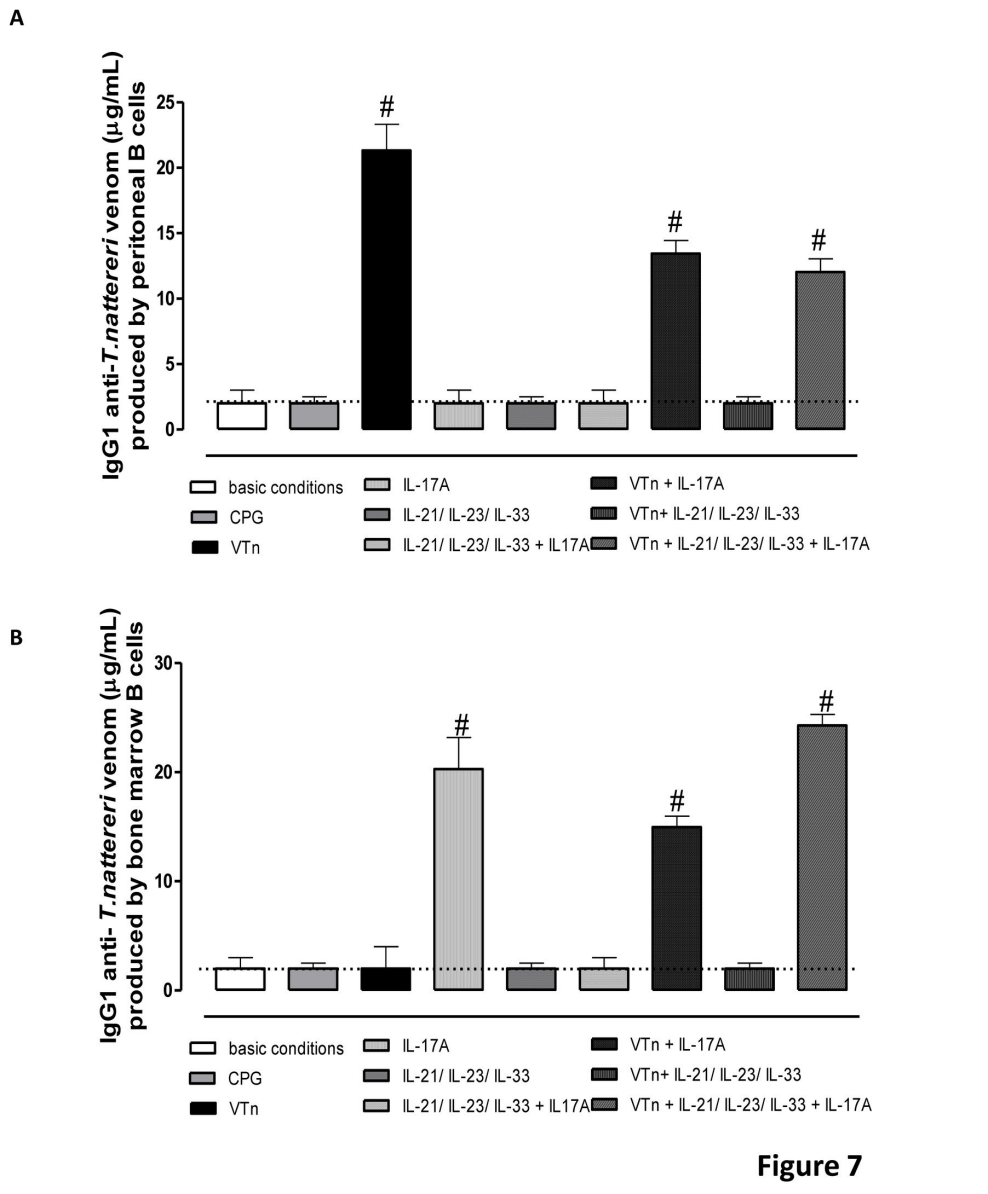
Venom and IL-17A control venom-specific IgG1 secretion by ASC. Purified CD19-positive B cells were cultured as described above. At the end of culture, ELISA harvested supernatants for quantifying Ab concentrations. Venom-specific IgG1 Abs were detected in supernatant of peritoneal (*A*) and BM (*B*) cell cultures. The dashed line represents the specific-IgG1 in supernatant of purified CD19-positive B cells from control group of mice cultured in medium under basic conditions. #*p* < 0.05 compared to CD19-positive B cells from V*Tn*-immunized mice in medium under basic conditions. Data are mean ± SEM values.

Abs secretion is the hallmark of terminal differentiated B cell [44]. To investigate whether differentiated CD138-positive ASC were functionally active we measured venom specific Ab secretion in the last day of culture. IgG1 was the predominant subclass secreted in supernatant from peritoneal or BM ASC, but specific IgG2a Abs were not detected (Figure **7**). These results show that V*Tn* acts increasing IgG1 secretion by CD138-positive ASC from peritoneal cavity of V*Tn*-immunized mice (Figure **7A**), while IL-17A is fundamental for stimulate the secretion of IgG1 by BM differentiated CD138-positive ASC (Figure **7B**). Our results show that the addition of IL-17A in venom-restimulated cells promoted a decrease in IgG1 production by peritoneal or medullar ASC.

Early studies demonstrated that IL-17A participates on antigen-specific Ig production since the efficient levels of Ig were reduced in mice deficient in IL-17 [25], and IL-17 together with BAFF, but not IL-17 alone increase cell survival, proliferation and Ig class switching via transcription factor Twist1 activation *in vitro* [45]. Milovanovic et al. [46] also demonstrated that IL-17A participates together with anti-CD40 and IL-4 in the IgE secretion by human ASC. Taken together, we demonstrate that activation of ASC for IgG1 secretion is triggered by venom proteins in peritoneal cavity and by the inflammatory cytokines as IL-17A maintained in medullar niche. Thus, the special retention of high-affinity Bmem in inflamed tissues and in central compartment as BM ensures that high-affinity Abs will be produced upon each Ag exposure.

## Discussion

The generation of vaccine-mediated protection is a complex challenge. The long-term protection requires the persistence of vaccine Abs and/or the generation of immune memory cells capable of rapid and effective re-activation upon subsequent microbial exposure. The determinants of immune memory induction, as well as the relative contribution of persisting Abs and of immune memory B cells to protection against specific diseases, are thus essential parameters of long-term vaccine efficacy. The successes in vaccines against polio, measles, smallpox, diphtheria and tetanus have mostly come against invariant pathogens that cause acute infections followed by long-term protective immunity. However, there are urgent needs to develop vaccines against persistent and chronic infections such as HIV, human papilomavirus, dengue, influenza, *Mycobacterium tuberculosis* and hepatitis C virus. Thus, a better understanding of how different antigens activate the immune system and sustain the immune memory is important for new vaccines and adjuvants or for the optimization of immunization strategies.

Here in this study, we confirm the contribution of Bmem to ASC differentiation. Using cellular suspensions of peritoneal cavity, spleen and BM from mice with chronic humoral response against venom (48 d), we purified switched CD19-positive Bmem that were cultured in an *in vitro* system in the presence of venom, cytokines or CpG. Together, our results confirm the existence of a hierarchic process of differentiation: activated memory B cells progressively acquire increasing levels of CD138 and decreasing levels of CD45R/B220 to finally arrive at ASC with B220^neg^ phenotype, which are IgG1-secreting cells. Only antigen-experienced Bmem from peritoneal cavity or bone marrow of V*Tn*-immunized mice presented the capacity to generate ASC functionally active, probably influenced by specific-niche stromal contact. This process is dependent on antigen and IL-17A itself. The reduction in the levels of CD45R/B220 and the increased expression of BAFF-R induced in ASC by IL-17A are both related to the direct action of this cytokine on Bmem in splenic and medullar niche. The differentiation of ASC induced by the venom is dependent on the BAFF-R signals and is independent on the Bcl-2 protein expression.

This work contributes for the expansion of the understanding of the factors involved in the differentiation and the survival of ASC, therefore it demonstrates that dependent on the microenvironment niche of their formation (mainly inflamed tissue as peritoneal cavity) these cells require the integration of signals derived from antigen and IL-17A for the survival for extending period of time and for the secretion of memory Abs. The trafficking and localization of Bmem and ASC in the body/tissue mediated by homing receptors and chemokine receptors triggered by venom antigens are determinant for activation dependent on BCR- or cytokine receptors.

Vaccines that induce neutralizing Abs have led to the eradication of important pathogens and have severely reduced the prevalence of many other infections. However, even the most successful vaccines do not induce protective Abs in all individuals, and can fail to induce lifelong immunity. In this view, our data permit some important points to be discussed. First, we demonstrated the capacity of proteins of the *T* *. nattereri* venom as B-cell helpers, leading committed Bmem to differentiation into IgG producing-ASC. The ability of venom antigens to promote the preferentially differentiation of Bmem from inflamed peritoneal cavity into IgG1-producing B220^neg^ ASC could be recognized as an adjuvant function for vaccines improvement, and highlight the important role of constant recruitment of new memory B cells for the continuously diversifying high-affinity Abs response produced upon each Ag exposure.

Second, our data show that IL-17A as an important mediator for memory immune responses, enhancing IgG Abs production and inducing IgG1 secretion lead us to suggest the use of IL-17A administration in combination with adjuvants as an immune-stimulator or the use of new adjuvants able to induce the local production of IL-17A. Our data corroborate the established concept that the generation of vaccine-induced Th17 cells as well IL-17 production is crucial for protection against intracellular organisms.

IL-17 has been increasingly implicated in host responses against intracellular pathogens, promoting the neutrophil infiltration and granulomatous inflammation at the site of infection. In addition, it has been attributed to IL-17 a role in antigen-specific Ig production with normal or impaired formation of GC [25,47-49]. Moreover, Th17 cells can induce B cell proliferation and promote antibody isotype switching to IgG1, IgG2a, IgG2b, and IgG3. Interestingly, IL-17 on its own drove class switch recombination to IgG2a [50].

Considerable recent attention has been given to IL-17 secreting CD4+ (Th17) cells and their potential role in vaccine-induced immunity to a diverse array of bacteria and viruses in preclinical models and several groups have recently reported that IL-17 confers protection against vaccine of *B. pertussis* [51], *C. albicans* or *Staphylococcus aureus* [52,53], systemic mycoses of North America, *B* *. dermatitidis* , *C* *. posodasii* , and *H* *. capsulatum* [54,55] and *S. Typhi* [56].

Finally, the potent activity of venom proteins to modulate innate immune cells. Emerging concepts suggest that information sensed about the antigen is integrated by dendritic cells (DC) and translated to antigen-specific T and B cells to modulate the strength, quality, and persistence of the memory immune response [57,58]. Moreover, conventional adjuvants, such as aluminum hydroxide, induce good Th2-type immune responses, but are not considered effective to promoting Th1-type immune responses. This is a major limitation in vaccines against pathogens for which potent cellular responses are required for protection, such as, respiratory syncytial virus (RSV), *Mycobacterium tuberculosis* and hepatitis C virus.

In this concept, venom proteins elicit both Th1- and Th2- memory immune responses with IL-17A production by T memory cells, and have even more potent activity in induce protective immunity, shaping the quantity and quality of T and B cell memory. Our group demonstrated recently that venom or isolated proteins modulate important checkpoints of an ideal vaccine antigen like co-stimulatory molecules on surface of antigen presenting cells, cytokine environment and memory cells. Nattectin, a C-type lectin isolated from the venom is able of overcoming the immaturity of the immune system driving Th1-type responses in an *in vivo* model and licenses macrophages to differentiate into cells exhibiting typical DC function *in vitro* [59]. Sustained production of cytokines (KC, IL-5, TNF-α, IL-6, IL-17A and IL-23) and leukocytes recruitment (neutrophils, eosinophils, and mast cells) were induced by venom which can enhance quality and quantity of effector and central memory T cell and ASC generation [13]. Moreover, proteases Natterins isolated from *T* *. nattereri* venom are also able to induce a pronounced Th2-type response and a rich splenic microenvironment important to generation and maintenance of terminal differentiated ASC with B220 negative phenotype [60].

In conclusion, the modulation of the capacity of specific-Bmem to differentiate into ASC could be achieved by a particular antigen and cytokines-based mechanisms; and is critical to fully explore the potential for design of novel vaccines or adjuvants in the future.

## Supporting Information

Figure S1 **Memory response induced by *T* *. nattereri* venom is characterized by high percentage of Bmem.** Dot plots (*A*) and percentage of Bmem (CD19^pos^ in B220^pos^ IgG^pos^ gated cells) in peritoneum (*B*), spleen (*C*) and bone marrow (*D*) from control- or V*Tn*-immunized mice were determined at 21, 28, 48, 74 and 120 d after immunization by multiparametric flow cytometry using Armenian hamster IgG1y2 FITC-anti-mouse CD19, goat IgG2bk PE-anti-mouse IgG (specific for IgG1, IgG2a, IgG2b and IgG3), Rat IgG2aak PerCP-Cy5-anti-mouse CD45R/B220. Data are mean ± SEM values from three independent experiments. *p < 0.05 compared to control-mice. Dot plots are representative of 3 experiments.
(TIFF)
